# Association of Endothelial Dysfunction and Antiretroviral Therapy in Early HIV Infection

**DOI:** 10.1001/jamanetworkopen.2019.13615

**Published:** 2019-10-18

**Authors:** Kelvin N. V. Bush, Julie L. Teel, James A. Watts, Rosco S. Gore, Gadiel Alvarado, Nathan L. Harper, Jason F. Okulicz

**Affiliations:** 1 Division of Cardiology, San Antonio Military Medical Center, San Antonio, Texas; 2 Infectious Disease Service, San Antonio Military Medical Center, San Antonio, Texas; 3 Foundation for Advancing Veteran’s Health Research, San Antonio, Texas

## Abstract

**Importance:**

Human immunodeficiency virus (HIV) infection is associated with increased cardiovascular disease (CVD) events. Endothelial dysfunction (EDF) is involved in CVD pathogenesis; however, EDF onset after HIV acquisition and the potential for reversibility with antiretroviral therapy (ART) have not been evaluated to date.

**Objective:**

To evaluate endothelial function with noninvasive reactive hyperemia index (RHI) in patients with early HIV infection at baseline and after ART initiation.

**Design, Setting, and Participants:**

Cohort study in which 61 members of the United States Air Force diagnosed with HIV infection from September 1, 2015, through September 30, 2017, were evaluated for baseline EDF. Natural log–transformed RHI values (lnRHI) of less than 0.51 and at least 0.51 were defined as abnormal and normal, respectively. The RHI interval changes were evaluated in a subgroup of 40 patients. Data were analyzed from September 30, 2017, through January 30, 2018.

**Exposure:**

Early HIV infection.

**Main Outcomes and Measures:**

Baseline EDF at HIV diagnosis and interval changes associated with ART initiation.

**Results:**

The 61 patients included in the analysis were predominantly male (58 [95%]) and mostly African American (35 [57%]), with a mean (SD) age of 28.1 (6.7) years at HIV diagnosis. Median time from estimated date of HIV seroconversion to RHI assessment was 10.6 months (interquartile range [IQR], 5.1-13.2 months), and the median CD4 lymphocyte count was 552/μL (IQR, 449/μL-674/μL). Patients had a mean (SD) body mass index of 26.2 (4.0), median (IQR) low-density lipoprotein cholesterol level of 97 (80-126) mg/dL, median (IQR) total cholesterol level of 163 (146-195) mg/dL, and no diabetes diagnoses. Overall mean (SD) lnRHI was 0.70 (0.29) at HIV diagnosis. Baseline RHI was normal in 47 patients (77%; mean [SD] lnRHI, 0.82 [0.20]) and was abnormal in 14 patients (23%; mean [SD] lnRHI, 0.30 [0.18]). Age (per 10-year increase) was not associated with an abnormal lnRHI (odds ratio, 2.15; 95% CI, 0.89-5.19; *P* = .09). Of the 41 patients with follow-up RHI assessments, 40 started ART immediately and repeated the RHI assessments at a median (IQR) of 6.4 (6.0-7.8) months. Use of ART was associated with an overall significan increase in mean (SD) lnRHI (0.13 [0.33]; *P* = .02). A greater increase in mean (SD) lnRHI was associated with abnormal (n = 11) compared with normal (n = 29) lnRHI at HIV diagnosis (0.33 [0.34]; *P* = .01 vs 0.04 [0.30]; *P* = .38). Among those with abnormal baseline lnRHI, 8 (73%) showed improved endothelial function after ART. The patient who declined ART converted from having a normal lnRHI (0.60) to an abnormal lnRHI (0.11) lnRHI after 8.3 months.

**Conclusions and Relevance:**

In this study, EDF was common in early HIV infection, with associated reversal in most patients taking ART. Results suggest that persistent EDF and CVD complications may be associated with delayed ART. Further studies are necessary to define the role of noninvasive endothelial function testing in patients with HIV infection.

## Key Points

**Question:**

Is endothelial dysfunction present in early HIV infection, and is reversal of endothelial dysfunction associated with antiretroviral therapy?

**Findings:**

In this cohort study of 61 patients with early seroconversion to HIV infection and low risk of cardiovascular disease, 14 had evidence of endothelial dysfunction. Antiretroviral therapy was associated with reversed endothelial dysfunction in 8 of 11 patients (73%) at follow-up.

**Meaning:**

Persistent endothelial dysfunction and subsequent cardiovascular disease may be associated with delayed initiation of antiretroviral therapy in patients with HIV infection, and reversal of endothelial dysfunction with antiretroviral therapy may be associated with mitigation of long-term complications of cardiovascular disease.

## Teaser

This cohort study examines endothelial function in patients with early HIV infection at baseline and after antiretroviral therapy initiation.

## Introduction

Individuals with HIV infection experience an increased burden of cardiovascular disease (CVD). Persons with HIV infection are twice as likely to develop CVD, and the incidence of HIV-associated CVD has tripled during the past 20 years.^1^ Risk factors for CVD are well described in the population with HIV but have not adequately accounted for the magnitude of CVD observed.^2^ Human immunodeficiency virus infection has been proposed to independently promote accelerated atherogenesis, vascular morphologic changes, and endothelial dysfunction (EDF).^3^ Modern risk assessment tools frequently underestimate CVD risk profiles and have not included substantial numbers of younger, asymptomatic patients with HIV.

Peripheral arterial tonometry (PAT) has gained increased interest as a tool to noninvasively assess endothelial function through measurement of pulsatile arterial volume changes by finger plethysmography (EndoPAT; Itamar Medical).^4^ Peripheral arterial tonometry has been validated in the Framingham Heart Study^4^ and Gutenberg Heart^5^ cohort to demonstrate peripheral finger endothelial function impairments correlating with coronary microvascular function and can be associated with cardiovascular end points. Prior PAT evaluation in patients with HIV infection has been limited, and the clinical role of noninvasive endothelial function testing has not been defined. This study examined baseline endothelial function by PAT in persons with early HIV infection and interval changes associated with antiretroviral therapy (ART) initiation.

## Methods

Members of the United States Air Force (USAF) on active duty who were diagnosed with HIV infection from September 1, 2015, through September 30, 2017 (n = 61), were included and retrospectively reviewed. All USAF members with incident HIV diagnosis were evaluated at the San Antonio Military Medical Center, San Antonio, Texas. Comprehensive cardiovascular evaluations with RHI were performed to assess personnel fitness for continued military duty. Clinical assessment with RHI determination was followed by immediate initiation of ART. A subgroup of patients (n = 41) had follow-up RHI assessments approximately 6 months after baseline. Natural log–transformed RHI values (lnRHI) of less than 0.51 and at least 0.51 were defined as abnormal and normal, respectively. These specific cutoff RHI values were adopted from manufacturer standards and prior studies^5,6^ that indicated peripheral EDF in populations with CVD. This retrospective analysis was approved by the institutional review board of San Antonio Military Medical Center, which waived the need for informed consent because the research involved no more than minimal risk to the participants and the waiver did not adversely affect their rights and welfare. This study followed the Strengthening the Reporting of Observational Studies in Epidemiology (STROBE) reporting guideline.

The RHI data were obtained at room temperature by a single licensed nurse practitioner (J.L.T.) using finger plethysmography (EndoPAT 2000). Each patient was seated comfortably for at least 15 minutes before testing. One PAT finger probe was placed on the index finger of the hand undergoing hyperemia testing, and a second PAT probe was placed on the contralateral index finger. A manual blood pressure cuff was placed on the upper arm of the tested limb 5 minutes before initiation of the examination for equilibration. The cuff was then inflated to a pressure of 50 mm Hg above the systolic pressure or 200 mm Hg for 5 minutes and then deflated to induce a reactive hyperemia. The PAT hyperemia ratio or RHI was defined as the ratio of the mean pulse-wave amplitude during the 1-minute period starting 60 seconds after cuff deflation divided by the mean pulse-wave amplitude of a 210-second preocclusion baseline period.

### Statistical Analysis

Clinical and demographic data were obtained by medical record review. Statistical analyses were conducted using unpaired *t* test, paired *t* test, Mann-Whitney test, and logistic regression analysis, with unpaired *P* < .05 indicating significance. Data were analyzed from January 30, 2017, through January 30, 2018.

## Results

The 61 patients included 58 men (95%) and 3 women (5%). Most patients were African American (35 [57%]). The mean (SD) age at HIV diagnosis was 28.1 (6.7) years (Table). The estimated date of seroconversion, calculated as the midpoint from the last HIV-negative test result and the first HIV-positive test result, was a median of 18.9 months (interquartile range [IQR], 8.1-24.4 months). The median (IQR) time from estimated date of seroconversion to baseline RHI assessment was 10.6 (5.1-13.2) months. At HIV diagnosis, the median (IQR) CD4 lymphocyte count was 552/μL (449/μL-674/μL) (to convert to ×10^9^ per liter, multiply by 0.001). Patients had a mean (SD) body mass index (calculated as weight in kilograms divided by height in meters squared) of 26.2 (4.0), median (IQR) total cholesterol level of 163 (146-195) mg/dL, and median (IQR) low-density lipoprotein cholesterol level of 97 (80-126) mg/dL at baseline (to convert cholesterol levels to millimoles per liter, multiply by 0.0259). Tobacco use was reported in 12 patients (20%), and no patients had diabetes.

**Table.** Characteristics at HIV Diagnosis and Cardiovascular Disease Risk Factors^a^

Characteristic	All (N = 61)	Normal lnRHI at HIV Diagnosis (n = 47)	Abnormal lnRHI at HIV Diagnosis (n = 14)	*P* Value^b^
Age, mean (SD), y	28.1 (6.7)	27.3 (5.6)	30.9 (9.4)	.19
Male, No. (%)	58 (95)	44 (94)	14 (100)	NA
Race, No. (%)				
African American	35 (57)	30 (64)	5 (36)	.12
White	20 (33)	13 (28)	7 (50)	
Other	6 (10)	4 (9)	2 (14)	
BMI, mean (SD)	26.2 (4.0)	26.0 (4.3)	26.9 (2.6)	.32
Hypertension, No. (%)	4 (7)	3 (6)	1 (7)	>.99
Tobacco use, No. (%)	12 (20)	9 (19)	3 (21)	>.99
Diabetes	0	0	0	NA
LDL cholesterol level, median (IQR), mg/dL	97 (80-126)	91 (78-125)	107 (86-138)	.39
Total cholesterol level, median (IQR), mg/dL	163 (146-195)	163 (146-195)	167 (149-199)	.82
Time from last negative HIV test to first HIV positive test, median (IQR), mo	18.9 (8.1-24.4)	16.8 (8.0-24.3)	22.8 (17.1-24.6)	.40
Time from estimated date of seroconversion to baseline lnRHI, median (IQR), mo	10.6 (5.1-13.2)	10.1 (5.0-13.1)	12.1 (9.4-13.4)	.35
CD4 lymphocyte count, median (IQR), No./μL	552 (449-674)	530 (436-673)	574 (483-670)	.98
HIV viral load, mean (SD) log_10_ copies/mL	4.4 (0.8)	4.4 (0.8)	4.6 (0.5)	.20
Baseline lnRHI, mean (SD)^c^	0.70 (0.29)	0.82 (0.20)	0.30 (0.18)	<.001

Abbreviations: BMI, body mass index (calculated as weight in kilograms divided by height in meters squared); IQR, interquartile range; LDL, low-density lipoprotein; lnRHI, natural log–transformed reactive hyperemia index; NA, not applicable.

SI conversion factors: To convert CD4 lymphocyte count to ×10^9^ per liter, multiply by 0.001; LDL and total cholesterol to millimoles per liter, multiply by 0.0259.

^a^ Percentages have been rounded and may not total 100.

^b^ Calculated using unpaired *t* test, paired *t* test, and Mann-Whitney test.

^c^ Calculated as the ratio of the mean pulse-wave amplitude during the 1-minute period starting 60 seconds after blood pressure cuff deflation divided by the mean pulse-wave amplitude of a 210-second preocclusion baseline period.

Overall mean (SD) baseline lnRHI was 0.70 (0.29) at HIV diagnosis. Forty-seven patients (77%) had a normal lnRHI (mean [SD], 0.82 [0.20]) and 14 (23%) had an abnormal lnRHI (mean [SD], 0.30 [0.18]). Age (per 10-year increase) and other demographic features, CVD profiles, and HIV disease characteristics were not significant. Follow-up RHI assessments were available for 41 patients (67%) at a mean of 6.4 months (IQR, 6.0-7.8 months) after baseline RHI. Forty of these patients (98%) initiated ART and 1 declined treatment. All regimens were anchored by integrase inhibitors dolutegravir (n = 28) or elvitegravir (n = 12) in combination with 2 nucleoside reverse transcriptase inhibitors. All patients receiving ART achieved viral suppression (viral load, <20 copies/mL) before the follow-up RHI assessment.

Early ART was associated with an overall significant increase in mean (SD) lnRHI (0.13 [0.33]; *P* = .02) (Figure). The mean (SD) increase in lnRHI was greater in those with an abnormal lnRHI (0.33 [0.34]; *P* = .01) compared with a normal lnRHI (0.04 [0.30]; *P* = .38) at baseline. Of the 11 patients with an abnormal baseline lnRHI, 8 (73%) achieved a normal lnRHI after initiating ART. No significant changes in body mass index or other CVD risk factors (such as cessation of tobacco use) were noted in those 8 patients. In addition, the patient who declined ART underwent conversion from a normal (0.60) to abnormal (0.11) lnRHI after 8.3 months of follow-up without ART.

**Figure.**
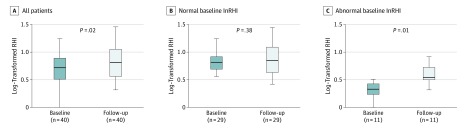
Boxplots Showing Natural Log–Transformed Reactive Hyperemia Index (lnRHI) The lnRHI was calculated as the ratio of the mean pulse-wave amplitude during the 1-minute period starting 60 seconds after blood pressure cuff deflation divided by the mean pulse-wave amplitude of a 210-second preocclusion baseline period. Data are shown at baseline and at follow-up overall and by normal lnRHI (≥0.51) or abnormal (<0.51) lnRHI at baseline. The center horizontal line indicates the median; the top and bottom borders of the box indiciate the third and first quartiles, respectively; and whiskers represent the maximum and minimum data points or interquartile range multiplied by 1.5 ± the third or first quartile.

## Discussion

Noninvasive endothelial function assessments continue to gain attention in preventive cardiovascular medicine efforts in the general and HIV-infected populations. Published rates of EDF are highly variable owing to different measuring techniques for endothelial function and the diverse populations studied. The RHI assessment was originally validated to detect coronary endothelial dysfunction, but recent studies^7^ suggest that RHI is more specific for the evaluation of peripheral vasculature. A meta-analysis by Matsuzawa et al^8^ evaluating 6 RHI studies of 1602 patients without HIV infection demonstrated that RHI was significantly associated with cardiovascular events. Ørbæk et al^9^ studied the correlation between RHI and myocardial flow reserve assessed by rubidium 82–labeled positron emission tomography and/or computed tomography in patients with HIV and found an inverse association between the 2 microvascular modality assessments. Their results could be explained by the differences in vascular physiological features that are exploited between the 2 examinations. The RHI has been validated using acetylcholine and nitric oxide flow–mediated dilation mechanisms. This method is in contrast to the myocardial flow reserve that is assessed by positron emission tomography and/or computed tomography with nitric oxide–independent adenosine or dipyridamole pharmacologic means. The study by Ørbæk et al^9^ ultimately highlights the variability of techniques to measure endothelial function and promotes the need for larger cohort studies that include patients with CVD.

Our study of endothelial function in young patients on active duty in the USAF represents a unique population with lowered cardiovascular risk profiles and early HIV infection. Sexual transmission has been described as the predominant mode of HIV acquisition in military personnel, whereas injection drug use is exceedingly rare owing to mandatory random drug testing.^10^ Our study reports nearly 25% of young persons with early HIV infection have noninvasive evidence of EDF by RHI. We believe that the RHI value substantiates formal design of EDF screening efforts in patients with HIV as a first step to identify patients at risk of CVD complications.

The natural history of endothelial function in HIV-infected persons remains poorly understood, and whether EDF is a consequence of HIV infection, long-term ART, or both in combination is uncertain. Infection with HIV is clearly associated with increased systemic inflammation and coagulation, both of which may be secondary to increased monocytes migrating across the endothelium and forming foam cells contributing to EDF.^11,12^ Antiretroviral therapy regimens, particularly the older-generation protease inhibitors, have been well described to promote dyslipidemia and participate in the pathogenesis of accelerated CVD.^8,9^ However, integrase inhibitor–based regimens have emerged as preferential options owing to the potency, tolerability, reduced metabolic effects,^10^ and new data suggesting decreased CVD risks.^11^

Endothelial dysfunction identified in clinical practice is managed with modification of cardiovascular risk factors, including weight loss, exercise prescriptions, angiotensin-converting enzyme inhibitors, lowering of low-density lipoprotein levels with statins, hormonal therapies, antioxidants, and smoking cessation.^12^ In our study, none of the patients received specific interventions beyond education and counseling. The patients in our study did not have significant changes in their body mass index, and no exercise prescriptions were ordered to suggest that changes in physical activity influenced the interval lnRHI changes. This leads us to conclude that ART initiation was significantly associated with the reversal of EDF observed after approximately 6 months of therapy. We believe this concept of ART associated with the improving endothelial function in our cohort with HIV is also strengthened by the 1 patient who declined ART and subsequently transitioned from a normal to abnormal lnRHI.

### Limitations

Limitations of our study include the small sample size and lack of availability of all patients for follow-up RHI testing. Despite these limitations, these findings merit further research to elucidate the incidence and natural history of EDF in persons with HIV infection and the effects of contemporary ART regimens.

## Conclusions

In this study, 23% of young patients with recent HIV seroconversion and low CVD risk had evidence of EDF. We observed that EDF reversed in association with early ART in most patients. Persistent EDF and associated CVD complications from HIV infection may be associated with delayed ART. Further studies are necessary to define the role and timing of noninvasive testing of endothelial function in patients with HIV infection.
